# Preventing Hypoxia in Pediatric MRI Sedation: A Randomized Controlled Trial of Prophylactic Nasopharyngeal Airway Placement

**DOI:** 10.7759/cureus.102861

**Published:** 2026-02-02

**Authors:** Jarupulla Parvathi, Santhosh Arulprakasam, Suryanarayanan Soundrarajan, Sakthirajan Panneerselvam, Priya Rudingwa, Aswini Kuberan, Satyen Parida

**Affiliations:** 1 Anaesthesiology and Critical Care, Mahatma Gandhi Medical College and Research Institute, Puducherry, IND; 2 Anaesthesiology and Critical Care, Jawaharlal Institute of Postgraduate Medical Education and Research, Puducherry, IND

**Keywords:** deep sedation, hypoxia, nasopharyngeal airway, pediatric anaesthesia, pediatric mri, propofol

## Abstract

Background: Deep procedural sedation for magnetic resonance imaging (MRI) is associated with airway collapse, causing hypoxia. The nasopharyngeal airway (NPA) is a simple rescue device, but there is limited literature on its prophylactic use to prevent hypoxia. This study evaluates the incidence of hypoxic episodes with and without prophylactic NPA placement in children undergoing MRI under sedation.

Methods: Two hundred and sixty-two American Society of Anesthesiologists (ASA) I and II children aged 1-10 years scheduled for MRI under sedation were randomized into the NPA and control groups. Both groups received intravenous midazolam, propofol bolus, and oxygen via a pediatric face mask. The NPA group had the airway placed, and sedation was maintained with propofol infusion. Hemodynamic, respiratory, and oxygen saturation parameters were monitored. Recovery time, discharge time, MRI quality, and adverse events were recorded.

Results: The NPA group showed a lower incidence of hypoxia (11.5% vs. 4.6%; P = 0.041) and faster recovery profile (5 (3.0, 6.0) vs. 5 (5.0, 7.0) minutes, P = 0.002). Propofol requirements (5 (3.6, 6.2) vs. 5 (4.1, 6.1) mg/kg, P = 0.539) and MRI duration (30 (25, 35 minutes) vs. 30 (25, 35 minutes), P = 0.226) were similar between groups. No major adverse events (epistaxis and laryngospasm) occurred in the NPA group. MRI quality was slightly better in the NPA group, though not statistically significant (P = 0.082).

Conclusion: Prophylactic NPA placement during propofol sedation for pediatric MRI reduces hypoxia, improves image quality, and minimizes interruptions. Sizing by nares-to-epiglottis distance ensures airway patency at multiple levels and, combined with capnography and oximetry, enhances the safety of deep sedation.

## Introduction

Magnetic resonance imaging (MRI) is a non-invasive diagnostic tool frequently used in children with various pathological conditions. However, successful MRI requires the child to remain motionless in a noisy, confined environment, necessitating sedation or anesthesia. The safety of procedural sedation hinges on maintaining a patent airway, adequate spontaneous ventilation, and stable hemodynamics. Children are particularly vulnerable to hypoxia and hypercarbia due to their higher metabolic demand and lower functional residual capacity compared to adults [1]. Anesthetic agents exacerbate this risk by reducing upper airway tone and depressing respiratory activity, leading to airway collapse during deep sedation.

Hypoxia and desaturation are the most common adverse events during procedural deep sedation, with reported incidences ranging from 1% to 14.8%. Hypoxia often necessitates urgent airway maneuvers such as bag-mask ventilation, which may lead to gastric insufflation and aspiration. It can also precipitate laryngospasm and bronchospasm due to lighter planes of anesthesia, thereby prolonging hospital stay and increasing morbidity [2-5]. Although several rescue airway devices are available, the nasopharyngeal airway (NPA) is advantageous as it maintains airway patency, is well tolerated during lighter planes of anesthesia and emergence, and is less traumatic compared to other invasive devices [6,7]. Prophylactic use of NPA may help prevent these complications and facilitate faster discharge following procedures under sedation.

We hypothesized that prophylactic NPA placement reduces airway collapse and hypoxia in children undergoing sedation for MRI. Accordingly, the primary objective of this study was to compare the incidence of hypoxic episodes between children with and without NPA placement. Secondary objectives were grouped to assess procedural efficiency (MRI scan interruptions and propofol requirements), airway characteristics and safety (NPA tip-to-epiglottis distance and adverse events), and recovery time.

## Materials and methods

This prospective randomized controlled trial was approved by the Institutional Ethics Committee (JIP/IEC/2019/0163, dated June 21, 2019) and registered with the Clinical Trials Registry of India (CTRI/2019/08/020721, dated August 8, 2019). Written informed consent was obtained from the parents or guardians of all participating children, and verbal assent was obtained from children above seven years of age. The study adhered to the 1975 Declaration of Helsinki and its later amendments, as well as the Consolidated Standards of Reporting Trials (CONSORT) guidelines. It was conducted from September 16, 2019, to August 18, 2021.

Participants

The study included 262 children aged 1-10 years, classified as American Society of Anesthesiologists (ASA) physical status I/II, scheduled for MRI under sedation. The patients were excluded if any of the following were present: (a) anticipated difficult airway, (b) macrocephaly due to hydrocephalus, (c) syndromic children with facial deformities, (d) clinical features suggestive of adenoid enlargement, and (e) congenital heart disease.

Randomization

Patients were allocated to either the NPA group or the control group using a computer-generated block randomization sequence with variable block sizes of 4 and 6, in a 1:1 allocation ratio, generated using SPSS version 19.0 (IBM Corp, Armonk, NY, US) by an independent research assistant. Consecutive sampling was employed to recruit eligible participants. Allocation concealment was ensured using sequentially numbered, opaque, sealed envelopes (SNOSE). Due to the nature of the intervention, this study was performed as an open-label trial.

Methodology

After confirming adequate fasting status, an intravenous (IV) cannula was secured. For younger, uncooperative children, a eutectic mixture of local anesthetic (EMLA) cream was applied to the venous cannulation site 30-60 minutes prior to cannulation. In the NPA group, nasal patency was assessed clinically. The appropriate NPA size was determined using the nares-to-epiglottic (NE) distance, calculated with the following formula:

$\text{NE distance} = 2.606 + 0.058 \times \text{Height (cm)} + 0.231 \times \text{NM distance} - 0.304 \times \mathrm{Gender}$

where NM distance was measured from the lateral border of the nose to the ipsilateral mandible angle and gender was coded as 0 for boys and 1 for girls [8].

To facilitate parental separation and patient positioning in the MRI suite, IV midazolam (50 µg/kg) was administered 10 minutes before the scheduled MRI scan to all the patients. Inside the MRI suite, patients were monitored using MRI-compatible three-lead electrocardiography with impedance tracing and pulse oximetry. End-tidal carbon dioxide (EtCO₂) was monitored using sidestream capnography via a specialized nasal cannula with an integrated sampling port, compatible with the MRI environment. Baseline heart rate, respiratory rate, oxygen saturation (SpO_2_), and EtCO_2_ were recorded. These parameters were monitored every minute for the first 10 minutes and subsequently every five minutes throughout the procedure. All children received oxygen supplementation via an appropriately sized facemask at a flow rate three times the minute ventilation to maintain a constant fraction of inspired oxygen (FiO_2_).

Both groups received titrated boluses of 1% propofol until loss of eyelash reflex, after which a lubricated, predetermined-size NPA was inserted with the head in neutral position in the NPA group only, while the control group proceeded directly to MRI without airway placement, and all children were positioned as per the radiologist requirements. Sedation was maintained in both groups with propofol infusion (1% propofol diluted with 5% dextrose to a concentration of 5 mg/mL) at 50-200 µg/kg/min using an IV flow connector regulator (Medi Tech Devices Pvt. Ltd, Ahmedabad, India). A qualified anesthesiologist continuously monitored vital parameters. Once adequate sedation was achieved (Ramsay Sedation Score (RSS) of 5 or 6), the MRI scan was initiated. The RSS is graded as 1: anxious, agitated, or restless; 2: cooperative, oriented, and tranquil; 3: responsive to commands only; 4: brisk response to light glabellar tap or loud auditory stimulus; 5: sluggish response to light glabellar tap or loud auditory stimulus; and 6: no response to glabellar tap [9].

After completing the MRI scan, propofol infusion was discontinued, and the child was transferred to the recovery area in the left lateral position. Recovery time was defined as the time taken to achieve an RSS of 2. MRI scan quality was assessed by the radiologist using a 5-point scale: 1: non-diagnostic; 2: poor quality with limited diagnostic value; 3: average; 4: good quality; and 5: excellent [10]. The tip of the NPA was marked, and its distance from the epiglottis was measured on the MRI image. The NPA was removed once the child was awake, responsive to commands, and showed no evidence of bleeding. Children were discharged upon achieving a modified Aldrete score of >9 [11].

If hypoxia (defined as a decrease in oxyhemoglobin saturation < 92%) occurred from airway obstruction (indicated by absent or decreased EtCO_2_ with evidence of respiratory effort), the anesthesiologist increased the oxygen flow rate, transiently reduced the propofol infusion rate, and alerted the radiology team to pause the MRI scan. The child was then removed from the MRI console, and airway maneuvers (e.g., head tilt, chin lift, jaw thrust, or oral airway placement) were performed to relieve obstruction. In the NPA group, the NPA position was rechecked.

If apnea (defined as the cessation of respiration for > 20 s) occurred, propofol infusion was immediately stopped, the child was removed from the MRI console, and bag-mask ventilation was initiated until spontaneous respiration and adequate SpO_2_ were restored. Appropriately sized laryngeal mask airways (LMA) and endotracheal tubes were kept available as rescue airway devices. Sedation failure was defined as the inability to complete the scan due to gross patient movement, the need for >3 repeated propofol boluses, or significant adverse events requiring supraglottic or tracheal airway placement under general anesthesia.

Sample size estimation

Sample size was calculated using OpenEpi (Open-Source Epidemiologic Statistics for Public Health, www.OpenEpi.com) for comparison of two independent proportions. Assuming an 8.6% incidence of hypoxia in the control group [4] and a 90% prevention rate in the experimental group, with 80% power, a 95% confidence interval (CI), and a 5% significance level, the required sample size was 250. Accounting for a 5% attrition rate, 262 participants (131 each) were planned.

Statistical analysis

Categorical variables were expressed as frequencies and percentages. Continuous variables were reported as mean (standard deviation) or median (interquartile range), based on normality assumptions. The Mann-Whitney U test was used for non-normally distributed data, and the unpaired t-test was used for normally distributed data. The Chi-squared test was used to compare gender distribution, ASA classification, incidence of hypoxia, interventions for hypoxia correction, MRI scan quality, total propofol dose, rescue propofol requirements, recovery time, discharge time, scan interruptions, and NPA tip position relative to the epiglottis between the control and NPA groups. Data were analyzed using SPSS version 19.0 (IBM Corp), with a P-value < 0.05 considered significant.

## Results

**Figure 1:**
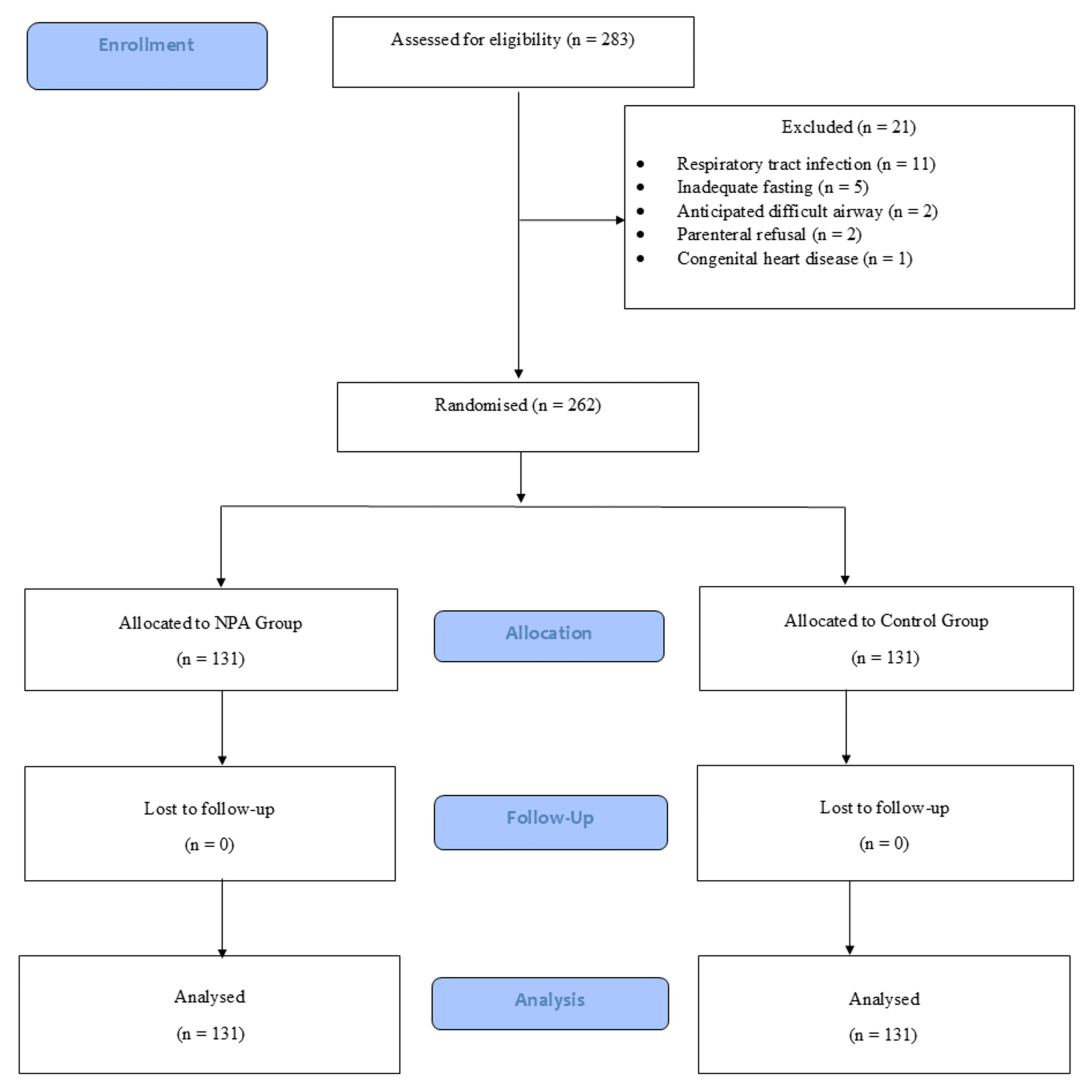
Consolidated Standards of Reporting Trials (CONSORT) flow of participants NPA: nasopharyngeal airway

Of the 283 children screened for eligibility, 262 participants were enrolled and randomized in Figure 1. Baseline characteristics were comparable and are summarized in Table 1.

**Table 1:** Demographic variables Values are depicted as median (interquartile range (IQR)) or number (proportion). *Mann-Whitney U test ^†^Chi-squared test NPA: nasopharyngeal airway; ASA: American Society of Anesthesiologists

Variable	Overall (n = 262)	NPA group (n = 131)	Control group (n = 131)	P-value
Age (years) (median (IQR))	3 (1.0, 5.0)	3 (1.0, 5.0)	2.5 (1.5, 5.0)	0.961*
Age group (%)				
Infant	70 (26.7)	39 (29.8)	31 (23.7)	0.239^†^
Toddler	87 (33.2)	37 (28.2)	50 (38.2)	
Child	105 (40.1)	55 (42.0)	50 (38.2)	
Weight (kg)	11.0 (8.0, 15.0)	11.0 (9.0, 15.0)	11.0 (8.0, 15.0)	0.811*
Gender (%)				
Boys	144 (55.0)	69 (52.7)	75 (57.3)	0.456^†^
Girls	118 (45.0)	62 (47.3)	56 (42.7)	
ASA (%)				
I	170 (64.8)	96 (73.3)	74 (56.5)	0.004^†^
II	92 (35.2)	35 (26.7)	57 (43.5)	

Outcome variables between the two groups were compared and presented in Table 2. Though hypoxia occurred in both groups, the incidence was 5% in the NPA group compared to 11.9% in the control group, corresponding to a relative risk of 0.4 (95% CI 0.1-0.9; P = 0.041). Interventions such as increasing oxygen flow and stopping propofol infusion (6.9% vs. 4.6%), chin lift and head tilt (1.5% vs. 0%), and jaw thrust (3.1% vs. 0%) were more frequently required in the control group than in the NPA group. The quality of MRI scans was better in the NPA group. Study interruptions occurred in 20 (15.3%) children in the NPA group and 35 (26.7%) in the control group, the predominant reasons being a child’s movement and hypoxia, both of which were more frequent in the control group (P = 0.47). Additionally, the NPA group demonstrated a better recovery profile (P = 0.002).

**Table 2:** Outcome variables Values are depicted as median (interquartile range) or number (proportion). *Mann-Whitney U test ^†^Chi-squared test NPA: nasopharyngeal airway; RR: relative risk; MRI: magnetic resonance imaging; CI: confidence interval

Variable	Total (n = 262)	NPA group (n = 131)	Control group (n = 131)	RR (95% CI)	P-value
MRI duration (min)	30 (27.0, 35.0)	30 (25.0, 35.0)	30 (29.5, 35.0)	-	0.226*
Desaturation (%)					
Not desaturated	241 (91.9)	125 (95.4)	116 (88.5)	0.4 (0.1, 0.9)	0.041^†^
Desaturated	21 (8.1)	6 (4.6)	15 (11.5)		
Intervention for desaturation (%)					
No intervention	241 (91.9)	125 (95.4)	116 (88.5)	-	0.50^†^
Stopped propofol and increased oxygen flow (%)	15 (5.8)	6 (4.6)	9 (6.9)		
Head tilt, chin lift (%)	2 (0.7)	0	2 (1.5)		
Jaw thrust (%)	4 (1.6)	0	4 (3.1)		
Interruption (%)					
Not interrupted	207 (79.0)	111 (84.7)	96 (73.3)	1.47 (1.0, 2.1)	0.02*
Interrupted	55 (21.0)	20 (15.3)	35 (26.7)		
Reason for interruption (%)					
Due to movement	47 (85.4)	18 (90.0)	29 (82.8)	-	0.47^†^
Due to hypoxia	8 (14.5)	2 (10.0)	6 (17.2)		
Recovery time (min)	5 (3.0, 6.0)	5 (3.0, 6.0)	5 (5.0, 7.0)	-	0.002*
Quality of MRI (%)					
Poor	3 (1.1)	2 (1.5)	1 (0.8)	-	0.082^†^
Average	35 (13.4)	16 (12.2)	19 (14.5)		
Good	166 (63.4)	76 (58.0)	90 (68.7)		
Excellent	58 (22.1)	37 (28.2)	21 (16.0)		

No significant differences were observed between the groups in terms of the median total propofol dose (P = 0.539) or the requirement for rescue propofol doses (P = 0.163; RR 1.22, 95% CI 0.9-1.6) (Table 3).

**Table 3:** Propofol rescue dose Values are depicted as median (interquartile range) or number (proportion). *Mann-Whitney U test ^†^Chi-squared test NPA: nasopharyngeal airway; RR: relative risk; CI: confidence interval

Variable	Overall (n = 262)	NPA group (n = 131)	Control group (n = 131)	RR (95% CI)	P-value
Requirement of rescue dose of propofol (%)					
Not given	192 (73.3)	101 (77.1)	91 (69.5)	1.22 (0.9, 1.6)	0.163^†^
Given	70 (26.7)	30 (22.9)	40 (30.5)		
Propofol dose (mg/kg)	5 (3.9, 6.1)	5 (3.6, 6.2)	5 (4.1, 6.1)	-	0.539*
Number of propofol doses (n = 71) (%)					
0	191 (72.9)	100 (76.3)	91 (69.5)	-	0.447^†^
1	58 (22.1)	26 (19.8)	32 (24.4)		
2	13 (5)	5 (3.8)	8 (6.1)		

**Figure 2:**
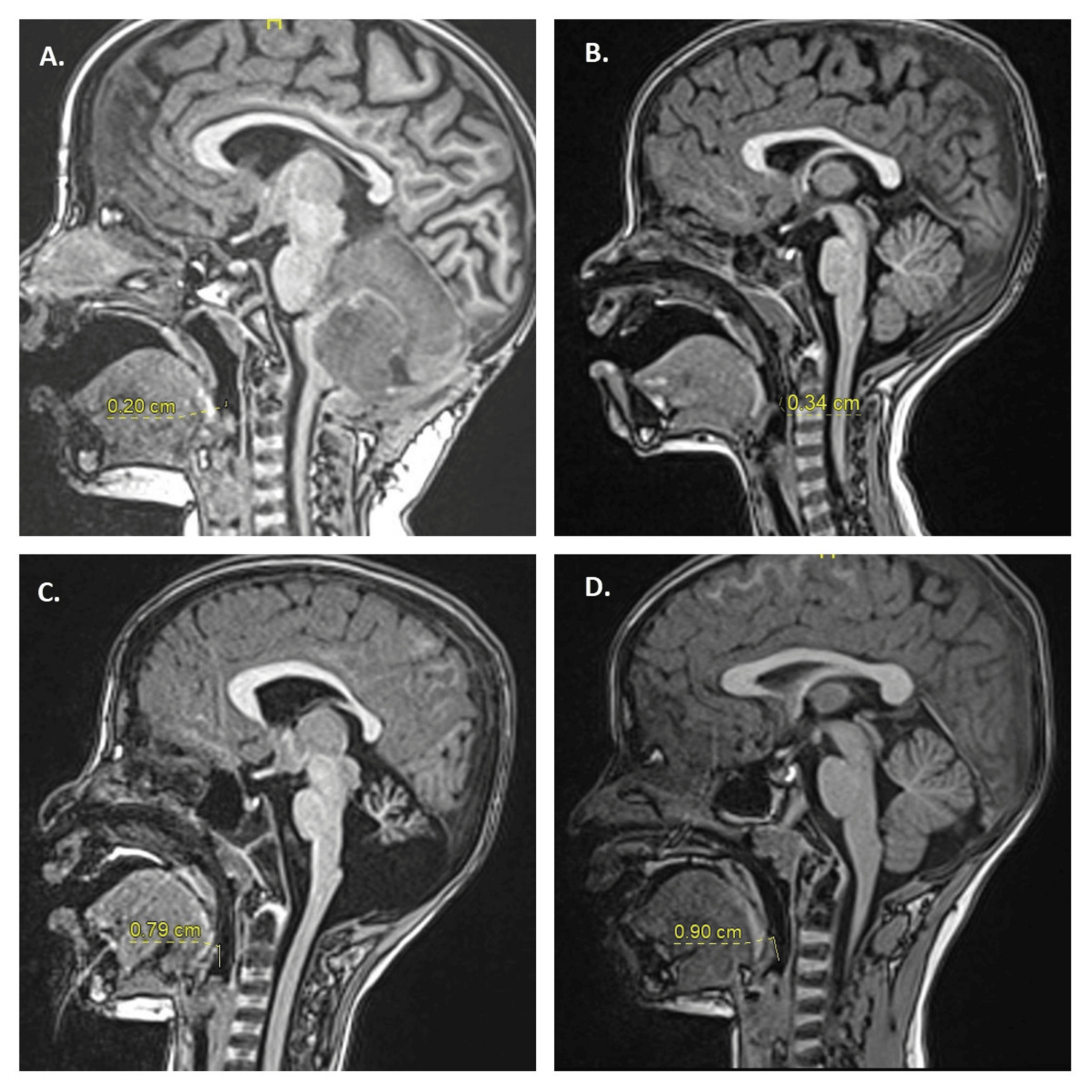
Magnetic resonance imaging (MRI) sagittal sections demonstrating the position of the nasopharyngeal airway (NPA) tip relative to the epiglottis NPA tip positioned above the epiglottis by 0.2 cm (A), above the tip of the epiglottis by 0.34 cm (B), below the tip of the epiglottis by 0.79 cm (C), and below the tip of the epiglottis by 0.9 cm (D).

The distance between the NPA tip and the epiglottis was also measured. In the NPA group, 106 (81%) participants had the NPA tip resting at or bypassing the epiglottis, eight (6%) had the tip extended beyond 1 cm from the epiglottis, 13 (10%) had the tip positioned above the epiglottis but within 1 cm, and four (3%) had the NPA tip anterior to the epiglottis, displacing it toward the posterior pharyngeal wall (Figure 2 and Table 4).

**Table 4:** Characteristics of NPA usage among the intervention group Values are depicted as frequency (percentage). NPA: nasopharyngeal airway

Variable	Frequency (n = 131)	Percentage (%)
NPA tip at epiglottis	116	88.5
Epistaxis	0	0
Blood staining of NPA	2	1.5
Cough	3	2.3

No major adverse events such as epistaxis was observed; however, blood staining of the NPA tip and coughing were noted in 1.5% (n = 2) and 2.3% (n=3) of cases (Table 4). There were no instances of sedation failure, LMA placement, tracheal intubation, or mechanical ventilation in either group.

## Discussion

The use of MRI has increased significantly; however, challenges associated with MRI include the claustrophobic and noisy environment, prolonged scan durations, and the need for MRI-compatible monitoring equipment. Direct observation and assessment of response to stimulation are difficult, as the child is positioned inside the MRI console, which can lead to interruptions, resulting in poor-quality images, the need for repeat scans, and resource wastage [12,13].

Hypoxia during propofol sedation has been reported at an estimated rate of 26.2 per 1,000 sedations [5], primarily due to the potent central respiratory depressant effects of propofol and its propensity to cause upper airway collapse by reducing upper airway tone [14,15]. While hypoxia secondary to hypoventilation can be mitigated through EtCO_2_ monitoring, careful titration of propofol infusion, and oxygen supplementation [16-18], hypoxia caused by airway collapse often necessitates physical airway maneuvers or adjuncts. Premedication with midazolam has been shown to significantly reduce both the induction and maintenance doses of propofol required for children aged 3-16 years undergoing sedation for MRI [19]. As part of a multimodal strategy to minimize hypoxia, all children received IV midazolam as a premedication to reduce propofol requirements.

Airway obstruction under sedation typically occurs at three levels: the soft palate, the base of the tongue, and the epiglottis [14,15,20,21]. Although traditional teaching attributes obstruction mainly to posterior displacement of the tongue, imaging studies using submandibular ultrasound have demonstrated a reduction in tongue thickness, while narrowing at the epiglottis and soft palate also contribute [15,19,20]. Thus, airway obstruction during sedation can occur at any of these levels. MRI-compatible capnography is invaluable in detecting hypoventilation and impending hypoxia before oxygen desaturation develops, and its role in ensuring safe sedation has been well established in multiple studies [14-16].

The NPA is a well-tolerated airway device available in various sizes and can be safely inserted even by non-anesthesiologists with minimal training. While its use as a rescue airway device is well established, its role as a prophylactic airway adjunct in pediatric sedation remains underexplored. Total propofol dose, procedure duration, and post-anesthesia care unit (PACU) time were significantly lower in children receiving NPA compared to LMA during flexible upper gastrointestinal endoscopy [22]. Our findings also indicated that the incidence of hypoxia was lower in children who received NPA immediately following propofol induction, likely owing to lower propofol requirements.

The ideal NPA length is closely related to age, weight, height, nares-to-mandible distance, and nares-to-tragus distance [8]. The significance of placing the NPA tip within 1 cm of the epiglottis, a finding that aligns with the majority of NPA placements, is consistent with the existing literature, as demonstrated in our study [23]. Notably, NPA length is more critical than diameter in maintaining upper airway patency [24].

Complications associated with NPA use are minimal, with epistaxis being the most concerning. Müller and his co-workers highlighted the benefits of routine NPA placement in adults undergoing endoscopy under propofol sedation, emphasizing the importance of NPA length in preventing airway collapse [25]. In our study, no episodes of epistaxis occurred during NPA insertion, and only two patients exhibited minor blood staining of the NPA.

Our study was designed to evaluate the benefits of prophylactic NPA use in the challenging MRI environment to ensure safe pediatric procedural sedation. Contrary to common concerns, placement of an NPA in children did not cause airway stimulation or bleeding in any participants. It effectively bypassed the soft palate, tongue base, and epiglottis, thereby maintaining airway patency and significantly reducing the incidence of desaturation, as hypothesized.

A few limitations include that the formula used to calculate the NE distance is complex and may not be practically feasible for all individuals undergoing MRI because of the time required for calculation. Fixed-length NPAs vary in length between manufacturers, complicating the choice of an appropriate size. Designing NPAs with adjustable flanges and standardized length markings could improve accuracy and clinical convenience. Furthermore, the radiologist assessing MRI quality could not be blinded, as the NPA was visible in the images. Similarly, the outcome assessor could not be blinded to group allocation.

## Conclusions

Prophylactic placement of an appropriately sized NPA, calculated using the NE distance, together with continuous monitoring of capnography and pulse oximetry, effectively maintained airway patency and reduced the incidence of hypoxia. This enhanced safety during deep sedation minimized procedural interruptions and improved image quality. Contrary to concerns regarding airway trauma in children, our findings suggest that prophylactic NPA use when inserted at the appropriate depth, with correct sizing, and at the right plane of sedation offers significant benefits, including fewer complications and smoother recovery.
